# Temporomandibular Joint Ankylosis among Patients at Saint Paul's Hospital Millennium Medical College, Ethiopia: A 9-Year Retrospective Study

**DOI:** 10.1155/2021/6695664

**Published:** 2021-02-17

**Authors:** Dereje Mekonnen, Andamlak Gizaw, Bruktawit Kebede

**Affiliations:** ^1^Department of Dental Oral and Maxillofacial Surgery, St. Paul's Hospital Millennium Medical College, Addis Ababa, Ethiopia; ^2^Department of Public Health, St. Paul's Hospital Millennium Medical College, Addis Ababa, Ethiopia

## Abstract

**Background:**

Temporomandibular joint ankylosis (TMJA) is a gradually developing pathological condition manifested by a limited mouth opening. It can result in an extremely disabling deformity that may affect mastication, swallowing, speech, oral hygiene, and facial cosmetic appearance. The present study aimed to determine the pattern of TMJA at St. Paul's Hospital millennium medical college (SPHMMC), Addis Ababa, Ethiopia.

**Methods:**

A retrospective descriptive study design was conducted at SPHMMC. All medical records of patients with the diagnosis of TMJA that visited the Maxillofacial Surgery unit from September 2010 through August 2019 were reviewed. Sociodemographic and clinical data including age, sex, place of residency, duration of TMJA cases, etiology, clinical presentations, imaging results, type of surgical operation, and complications after surgery were collected and analyzed using IBM SPSS software version 20 for Windows (Armonk, NY, USA: IBM Corp) computer program.

**Results:**

A total of 130 patients' medical records were reviewed. Out of this, 95 were included in the study. Forty-two (44.2%) of the TMJA cases were males, while the remaining 53 (55.8%) were females with a male to female ratio of 0.79 : 1. 20–29-year-old patients were the most affected, 36 (37.9%), followed by the 30 to 39 years age group, 33 (34.7%). Trauma (77.9%) was identified as the most common cause of TMJA. Notably, bilateral ankylosis (72.6%) was more common than unilateral (27.3%), and micrognathia was the most common (23.0%) deformity observed. The majority 52 (54.7%) of TMJA patients were treated with gap arthroplasty.

**Conclusions:**

TMJA was predominant among females than their male counterparts. Of note, 20–29-year-old patients were the most affected group. The majority of TMJA cases were treated by gap arthroplasty with almost no postoperative complications. Early detection and intervention to release the ankylosed joint is needed to improve patients' quality of life.

## 1. Background

Temporomandibular joint (TMJ) is a synovial diarthrodial joint that is formed between the condyle of the mandible and the glenoid fossa of the temporal bone that are separated by an articular disc [1]. Temporomandibular joint ankyloses (TMJA) implies a clinical condition characterized by the fusion of the mandibular condyle to glenoid fossa in the base of the skull [2, 3]. Classifications of TMJA fall into different groups based on the number of joints involved as unilateral or bilateral; location as intra-articular or extra-articular; type of tissue involved as bony, fibrous, or fibro-osseous, and extent of fusion as complete or incomplete [1, 4]. Besides, Sawhney classified TMJA into four groups as type I, II, III, and IV according to radiography results [5].

TMJA is frequently associated with trauma, mainly condylar fracture [6], infection (middle ear infection) [7], cancrum oris [8], septic arthritis [9], and systemic inflammatory conditions such as ankylosing spondylitis, rheumatoid arthritis, and psoriasis [10, 11]. Its clinical presentations depend on the age at which ankylosis occurs, the duration of TMJA, and whether the ankylosis is unilateral or bilateral. When it occurs in children before growth has ceased, it presents with serious and disabling problems of mastication, digestion, speech, appearance, and oral hygiene [12, 13]. Consequently, it will affect the nutritional status, psychosocial development, growth, and development of the jaws and teeth, as well as the oral hygiene status of the child [4, 14]. But, when it occurs after the growth has ceased, patients present mainly with limited mouth opening [10].

The diagnosis of TMJA can be made using clinical history and physical examination. Besides, TMJ imaging can be used to appreciate the anatomy of the joint, to classify the ankylotic mass based on the tissue content and the anatomical structures involved. Therefore, imaging such as plain radiography, panoramic, CT scan, arthrography, three dimensional CT scan, MRI, ultrasonography, and radionuclide imaging could enhance the proper diagnosis of TMJ ankyloses [2, 15]. The management of TMJA is mainly through surgical intervention [1, 16]. Therefore, it should be initiated as soon as the condition is recognized, with the main objective of reestablishing joint function, harmonious jaw function, and improve the patient's quality of life [17, 18]. The present study aimed to assess the pattern of TMJA at St. Paul's Hospital Millennium Medical College (SPHMMC), Addis Ababa, Ethiopia. The hospital has been chosen for this study because its Maxillofacial Unit is one of the pioneer centers of excellence that provides services related to oral and maxillofacial health conditions including TMJA cases in Ethiopia.

## 2. Methods

A retrospective descriptive study design was conducted at SPHMMC, Addis Ababa, Ethiopia, from September 2010 to August 2019. All patients with the diagnosis of TMJA and complete medical records were included in the study. Cases with incomplete medical records were excluded. Data such as age, sex, place of residency, duration of the case, etiology, clinical presentations, imaging results, type of operation, and complications after surgery were retrieved from medical records of the patients using a structured checklist developed by reviewing previously published articles. The collected data were analyzed by using IBM SPSS software version 20 for Windows (Armonk, NY, USA: IBM Corp). Ethical clearance was obtained from the Research and Ethical committee of SPHMMC, and permission was sought from SHMMC board before the study was conducted.

## 3. Results

### 3.1. Sociodemographic Characteristics

A total of 130 patient's medical records with the diagnosis of TMJA were reviewed. Of these, 95 (73.1%), of the records fulfilled the inclusion criteria and were included in the analyses. From a total of 95 reviewed medical records, 53 (55.8%) were females, while the remaining 42 (44.2%) were males. The majority, 36 (37.9%), of patients were within the age group of 20–29 years with a mean age of 19.6 years (SD ± 10.31). Sixty-one (64.1%) of the patients were living in rural areas of the country (Table 1).

### 3.2. Causes and Duration of TMJA

**Figure 1:**
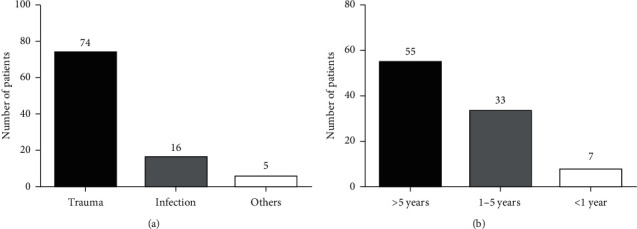
Causes and duration of TMJA. The causes (a) and time from the onset to the presentation of patients to the clinic (b).

The causes of TMJA included trauma 74 (77.9%), infection 16 (16.8%), and unknown 5 (5.3%) (Figure 1(a)). Trauma was the most common cause of TMJA in 41 female and 32 male patients. TMJA caused by trauma was predominant among 20–29-year-old patients that accounted for 29 (30.5%) of the cases. On the other hand, 11 (11.6%) TMJA cases were caused by infection among patients of 30–39 years of age. Of these, three patients had a childhood middle ear infection, four open wound infections, and the rest four unspecified infections. From a total of 73 TMJA cases caused by trauma, 46 of the patients reside in rural areas (Table 2).

The time from the onset to the presentation of patients to our clinic was more than five years in 55 (57.9%) of TMJA cases followed by 1–5 years in 33 (34.7%) and less than one year in 7 (7.4%) of the patients (Figure 1(b)).

### 3.3. Classes of TMJA and Affected Sides

From the total of 95 TMJA cases, 69 (72.6%) were bilateral and the rest 26 (27.4%) were unilateral (Table 3). Among the unilateral cases, 16 (16.8%) were on the right side while the remaining 10 (10.5%) were on the left side. Eighty-five (89.5%) of the TMJA cases were bony type, while the remaining 10 (10.5%) were fibrous type. From the total of 85 bony TMJA cases, 64 (75.3%) of them were bilateral, and the rest 21 (24.7%) were unilateral where 12 (12.6%) and 9 (9.4%) of them were seen on the right and left side of TMJ, respectively. Of the 10 (10.5%) fibrous TMJA cases, 5 (5.2%) were bilateral and the rest 5 (5.3%) were unilateral fibrous type of TMJA (Table 3).

### 3.4. Clinical Presentations of TMJA

TMJA patients present with different clinical manifestations. Clinical presentations of the patients include oral manifestations such as poor oral hygiene, rampant caries, periodontal problem, cross bite, class II malocclusion 80 (23.6%), micrognathia 79 (23.0%), antegonial notch 65 (19.2%), bird face 61 (18.0%), nil mouth opening 28 (8.3%), and deviation to the affected side 26 (7.7%) (Table 4).

### 3.5. Surgical Treatment and Its Complications

From a total of 95 patients, 58 (61.05%) of the TMJA cases were treated with gap arthroplasty (GA), while 37 (38.95%) were treated with interpositional arthroplasty (IA) in addition to GA (Table 5). There were reports of only three complications due to the surgical treatments undertaken. Notably, two of the patients had reankylosis, and one patient had an infection. Surgical treatments were considered successful when patients had a minimum of 3.5 cm interstitial mouth opening postoperation. All patients had follow-up every month for the first six months and every three to six months for an additional one and a half years.

## 4. Discussion

Temporomandibular joint ankylosis (TMJA) is a debilitating health condition that causes difficulty in mastication, speech, and mouth opening, and it can be debilitating if left untreated [11]. Its management requires careful surgical intervention plans. In this study, we described the pattern of TMJA in one of the tertiary-level hospitals in Ethiopia over a period of nine years. TMJA cases were predominant among females than males with a male to female ratio of 0.79 : 1. Notably, trauma was identified as the most common cause of TMJA in 74 (77.9%) of patients.

In the present study, ankylosis cases were more predominant in females (55.8%) than males (44.2%), with a male to female ratio of 0.79 : 1. This finding is in agreement with other reports from Sudan [2], Morocco [19], South Africa [10], Pakistan [20], and Nigeria [21]. In contrast, studies from Nigeria [4] and India [18] reported the predominance of TMJA cases among males than females.

In this study, TMJA had a wide age range distribution ranging from 4 to 65 years, with a mean age of 19.6 years. It was frequently seen in the 3rd and 4th decades of life. This finding is similar to a report from Morocco [19] in which peak age was the 3rd decade of life. On the contrary, a report from Indonesia [13] revealed the 2nd decade of life as a peak age.

The pathogenesis of TMJA can either be primary, when the pathological process directly affects the TMJ, as in the case of systemic diseases such as ankylosing spondylitis, rheumatoid disease, and psoriasis. or secondary, as in traumatic injury, which may cause intracapsular condylar fractures and heamarthrosis [22, 23]. Trauma was identified as the most common cause of TMJA among the patients accounting for about 77.9% of the cases. This finding is consistent with reports from different parts of the world, such as Sudan [2], India [3], China [17], Indonesia [13], Turkey [16], and Pakistan [20] that reported trauma as the most common cause of TMJA. The higher number of trauma-caused TMJA cases might be because a large number of patients (64.2%) were from rural areas where dental services in general and maxillofacial surgery services in particular are still in infancy in Ethiopia. As a result, most of the patients with jaw fractures might have either remained undiagnosed or are managed unsatisfactorily.

TMJA cases could present as a bilateral or unilateral lesion based on the number of joints affected. Most (72.6%) of the patients presented with bilateral TMJA. This finding is similar to studies from Sudan [2], China [24], and India [25]. The high prevalence of bilateral TMJA might be due to the trauma (falling) mechanism that could result in mandibular symphysis fracture which, in turn, produces a higher chance of osteogenic potential bone fragments in the condylar process. On the other hand, our finding is inconsistent with a report from Pakistan [20] that reported more cases of unilateral than bilateral TMJA. Among unilateral patients, most cases were seen on the right side than the left side, a finding consistent with reports from Sudan and Pakistan [2, 20]. However, a study from China reported a higher number of TMJA cases on the left side than the right side [24].

In the present study, majority of TMJA cases presented to our clinic after a significant delay, more than five years, from the onset of their symptoms. This finding is consistent with a report from Sudan [2]. The delay in the presentation of the patients might relate to the place of residence of the patients where most of them live in rural areas that have limited access to early treatment. Furthermore, lack of awareness and poor socioeconomic status could potentially attribute to the higher number of TMJA [26].

TMJA is one of the complex health problems that could result in micrognathia, difficulty in mastication and swallowing of food and speaking, and poor oral hygiene leading to dental caries and periodontal diseases [27]. In the present study, our TMJA patients were presented with micrognathia (23.0%), poor oral hygiene (23.6%), antegonal notch (19.2%), nil mouth opening (8.3%), and deviation of the chin to the affected side (7.7%). This finding is in line with reports from India [3], Morocco [19], and South Africa [10]. Thus, early detection and surgical intervention to release the ankylosed joint will improve the patient's quality of life.

The management of TMJA mainly relies on surgical procedures such as gap arthroplasty (GA), interpositional arthroplasty (IA), and reconstruction of the articulation (RA) to restore the function of joint and prevent reankylosis [17]. GA involves removal of a block of bone, either the complete condyle or a full-thickness section of bone, leaving a minimum of 1 cm gap between the ascending ramus and the temporal bone to prevent reankylosis [28]. IA is a modification of GA that involves the insertion of interpositional materials (autogenous or alloplastic) into the space created by GA to prevent reankylosis [17]. In the present study, 54.7% of the patients were treated with GA, while the remaining 35.8% had IA in addition. Importantly, only two patients had reankylosis after six months of treatment. This finding is consistent with a meta-analysis study that showed no significant difference in the incidence of reankylosis between the IA and the GA [17]. The reason for reankylosis might be due to inadequate removal of the bone between the ascending ramus and temporal bone or wound infection at the surgical site or poor compliance of the patients for postoperative physiotherapy [29]. The present study had limitations. As the study followed a hospital-based retrospective study design, incomplete medical records that rendered to the exclusion of a quarter of cases from the analysis might have introduced a selection bias.

## 5. Conclusions

In the present study, TMJA was predominant among females than their male counterparts. 20–29-year-old patients were the most affected group. Of note, trauma was identified as the most common cause of TMJA. Majority of patients were surgically treated with gap arthroplasty (GA). Importantly, only three of the patients had complications after surgical treatments, of which two patients had reankylosis and one patient had an infection. Thus, early detection and surgical intervention to release the ankylosed joint improves the patient's quality of life.

## Figures and Tables

**Table 1:** Sociodemographic characteristics of the patients.

Sociodemographic status		Frequency (%)
Age (years)	0–9	6 (6.3)
	10–19	14 (14.7)
	20–29	36 (37.9)
	30–39	33 (34.7)
	≥40	6 (6.3)

Sex	Male	42 (44.2)
	Female	53 (55.8)

Residency	Rural	61 (64.2)
	Urban	28 (29.5)
	Not recorded	6 (6.3)

**Table 2:** Sociodemographic characteristics and causes of TMJA.

Sociodemographic status		Causes			Total
		Trauma	Infection	Others	
Sex	Female	41	7	5	53
	Male	32	9	1	42

Age (years)	0 to 9	5	0	1	6
	10 to 19	11	1	2	14
	20 to 29	29	4	3	36
	30 to 39	22	11	0	33
	>40	6	0	0	6

Residency	Rural	46	12	3	61
	Urban	21	4	3	28
	Not recorded	6	0	0	6

**Table 3:** Frequency distribution of TMJA classes based on the affected side and tissue type.

Affected side	Tissue		Total frequency (%)
	Bony frequency (%)	Fibrous frequency (%)	
Bilateral	64 (67.4)	5 (5.2)	69 (72.6)
Right unilateral	12 (12.6)	4 (4.2)	16 (16.8)
Left unilateral	9 (9.4)	1 (1.1)	10 (10.5)
Total	85 (89.5)	10 (10.5)	95 (100)

**Table 4:** Frequency distribution of clinical presentation of the patients.

Clinical presentation	Frequency (%)
Oral manifestation	80 (23.6)
Micrognathia	79 (23.0)
Antegonal notch	65 (19.2)
Bird face	61 (18.0)
Nil mouth opening	28 (8.3)
Deviated to the affected side	26 (7.7)

**Table 5:** Frequency distribution of surgical treatments used in the management of patients.

Type of treatment		Frequency (%)
Gap arthroplasty (GA)		58 (61.05)
Interpositional arthroplasty (IA)	Temporalifacial flap	34 (35.8)
	Abdominal fat	2 (2.1)
	Custom-made prosthesis	1 (1.1)
Total		95 (100)

## Data Availability

The row data for this study are available on request from the corresponding author.

## Abbreviations

SPHMMC: St. Paul's Hospital Millennium Medical College
TMJ: Temporomandibular joint
GA: Gap arthroplasty
IA: Interpositional arthroplasty
RA: Reconstruction of the articulation
